# Type IV Pilus-Mediated Inhibition of *Acinetobacter baumannii* Biofilm Formation by Phenothiazine Compounds

**DOI:** 10.1128/spectrum.01023-23

**Published:** 2023-06-21

**Authors:** Nam Vo, Benjamin S. Sidner, Yafan Yu, Kurt H. Piepenbrink

**Affiliations:** a Department of Biochemistry, University of Nebraska-Lincoln, Lincoln, Nebraska, USA; b Department of Food Science and Technology, University of Nebraska-Lincoln, Lincoln, Nebraska, USA; c Department of Chemistry, University of Nebraska-Lincoln, Lincoln, Nebraska, USA; d Nebraska Food for Health Center, University of Nebraska-Lincoln, Lincoln, Nebraska, USA; e Center for Integrated Biomolecular Communication, University of Nebraska-Lincoln, Lincoln, Nebraska, USA; Griffith University

**Keywords:** *Acinetobacter*, biofilm, phenothiazine, thioridazine, type IV pili

## Abstract

Infections by pathogenic Acinetobacter species represent a significant burden on the health care system, despite their relative rarity, due to the difficulty of treating infections through oral antibiotics. Multidrug resistance is commonly observed in clinical Acinetobacter infections and multiple molecular mechanisms have been identified for this resistance, including multidrug efflux pumps, carbapenemase enzymes, and the formation of bacterial biofilm in persistent infections. Phenothiazine compounds have been identified as a potential inhibitor of type IV pilus production in multiple Gram-negative bacterial species. Here, we report the ability of two phenothiazines to inhibit type IV pilus-dependent surface (twitching) motility and biofilm formation in multiple Acinetobacter species. Biofilm formation was inhibited in both static and continuous flow models at micromolar concentrations without significant cytotoxicity, suggesting that type IV pilus biogenesis was the primary molecular target for these compounds. These results suggest that phenothiazines may be useful lead compounds for the development of biofilm dispersal agents against Gram-negative bacterial infections.

**IMPORTANCE** Acinetobacter infections are a growing burden on health care systems worldwide due to increasing antimicrobial resistance through multiple mechanisms. Biofilm formation is an established mechanism of antimicrobial resistance, and its inhibition has the potential to potentiate the use of existing drugs against pathogenic Acinetobacter. Additionally, as discussed in the manuscript, anti-biofilm activity by phenothiazines has the potential to help to explain their known activity against other bacteria, including Staphylococcus aureus and Mycobacterium tuberculosis.

## INTRODUCTION

Acinetobacter species are nonflagellated, Gram-negative coccobacilli which can be found in a wide range of terrestrial or aquatic environments but also form commensal or pathogenic relationships with human hosts. Acinetobacter baumannii, in particular, is known to cause infections in the lungs, skin, urinary tract, and blood, and many infections are also caused by the closely related A. calcoaceticus, A. nosocomialis, and A. pittii (the Acinetobacter calcoaceticus*-baumannii* or Acb complex) (1). The CDC 2019 antibiotic resistance threat report classifies carbapenem-resistant Acinetobacter (CARB), regardless of species, as an urgent threat because of the difficulty of treating these multidrug-resistant infections (2). Because of its multidrug resistance, A. baumannii is also included in the list of ESKAPE pathogens (Enterococcus faecium, Staphylococcus aureus, Klebsiella pneumoniae, Acinetobacter baumannii, Pseudomonas aeruginosa, and Enterobacter spp.) (3). Much like another ESKAPE pathogen, P. aeruginosa, most Acinetobacter infections are nosocomial, being most commonly associated with the use of ventilators or catheters (4).

One critical factor in the epidemiology of Acinetobacter infections and the difficulty of treating them is the ability of Acinetobacter to form biofilms on biotic (host tissue) and abiotic (metal or plastic) surfaces. Biofilms formed on medical devices can be a source of transmission from patient to patient, and biofilms allow for both persistent and recurrent infections in health care settings. Bacterial biofilms are multicellular structures composed of bacterial cells encased in an extracellular matrix of polypeptides, polysaccharides, and DNA. Bacterial biofilms form an encapsulating barrier against host or environmental factors, making cells within a biofilm more resistant to antibiotic compounds than planktonic cells.

**FIG 1:**
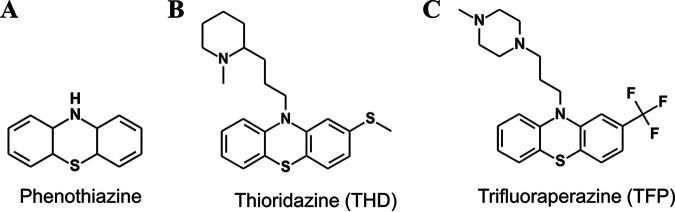
Phenothiazine compounds. Chemical structures of (A) phenothiazine and two derivatives, (B) thioridazine (THD) and (C) trifluoperazine (TFP).

Bacterial biofilm formation is a multi-factorial process with numerous secreted proteins (5), extracellular appendages (6–9), and quorum-sensing mechanisms (10) implicated in various species. In Acinetobacter, our group previously found that type IV pili are essential for robust biofilm formation in vertical biofilm assays (11). Type IV pili (T4P) are helical filaments comprised of protein subunits (pilins) stabilized by the association of hydrophobic N-terminal alpha helices in the core of the pilus fiber (12, 13). They can be extended or retracted through complex membrane-spanning machinery, allowing them to function in adhesion, DNA uptake (14, 15), and surface motility (16) in numerous Gram-negative and Gram-positive species. Twitching motility, a form of surface motility which can occur at the interface between solid and semisolid surfaces, is mediated solely by T4P (17–20) and requires both efficient extension and retraction of the pilus (16, 21, 22). T4P are well-characterized in *Neisseria*, where they are essential for the formation of microcolonies on host cells (23, 24). To inhibit host adhesion by *Neisseria*, Denis et al. screened for inhibitory compounds and found two structurally related compounds which acted specifically through the inhibition of T4P in Neisseria meningitidis (25). These two compounds, trifluoperazine (TFP) and thioridazine (THD), belong to the family of phenothiazine derivatives or phenothiazines (Fig. 1).

Phenothiazines are a class of heterocyclic compounds with a variety of applications and a widespread history of use in biology and medicine. Derivatives of phenothiazine have been used as histological stains (26), sedatives (27), antipsychotic agents (28), and antimicrobials (29, 30). One piperidine, thioridazine, has been shown to have activity against both extensively drug-resistant Mycobacterium tuberculosis (31, 32) and methicillin-resistant S. aureus (MRSA) (33–35). For both of these organisms, the primary therapeutic effect has been the potentiation of other antimicrobial compounds due to diminished capacity for microbial resistance.

The phenothiazine compounds previously found to inhibit *Neisseria* microcolony formation were also found to inhibit T4P-dependent functions in P. aeruginosa in the same study, suggesting that they may have broad activity against Gram-negative T4P systems, including those in Acinetobacter. Here, we report the activity of phenothiazine compounds against T4P-dependent functions, including biofilm formation, in A. baumannii and other Acinetobacter species.

## RESULTS

### Thioridazine and trifluoperazine are well-tolerated by *A. baumannii* and *P. aeruginosa*, but not by *Moraxella bovoculi*, at μM concentrations.

To gauge the suitability of phenothiazines as inhibitors of type IV pilus production in Acinetobacter and related genera, we first used planktonic growth in the presence of working concentrations of two phenothiazines to measure bacteriostatic or bacteriotoxic activity. For species where these compounds are cytotoxic, inhibition of specific functions (twitching motility or biofilm formation) might be difficult to disentangle from cytotoxicity. Previous studies of phenothiazines as antimicrobials against S. aureus, N. meningitidis, and M. tuberculosis used concentrations ranging from 2 to 120 μM, with MIC values as low as 20 μM depending on the species in question (25, 32, 35). Most recently, to inhibit T4P function in *Neisseria*, Denis et al. (25) used concentrations of two phenothiazine-derivatives, thioridazine and trifluoperazine (Fig. 1), at concentrations ranging from 10 to 40 μM, observing effects on T4P function in as little as 30 min and growth defects after approximately 2 h.

To compare the bactericidal or bacteriostatic activity of THD and TFP against Acinetobacter to these previous observations, we chose concentrations of 10 and 50 μM, encompassing the range where both inhibition of T4P function and potentiation of other antimicrobials were observed. We measured the growth in broth of three *Pseudomonadales* species, Acinetobacter nosocomialis M2, P. aeruginosa PAO1, and Moraxella bovoculi 58086, at 10 and 50 μM THD, as well as A. nosocomialis and *M. bovoculi* at 10 and 50 μM TFP. Over 12 h, A. nosocomialis and P. aeruginosa showed nearly identical optical density of bacterial cultures at 0, 10, and 50 μM THD. A. nosocomialis also showed no substantial growth defect at 10 or 50 μM TFP over the observed time course (6 h). However, both compounds significantly limited the growth of M. bovoculi at 10 μM. For each curve (Fig. S1 in the supplemental material), bacteria in 96-well plates were incubated at 37°C, shaken every 30 minutes, and optical density was measured immediately afterward. Due to the cytotoxicity of TFP and THD against *M. bovoculi*, further investigations used Acinetobacter and *Pseudomonas* species.

### Phenothiazines inhibit twitching motility in *Acinetobacter*.

**FIG 2:**
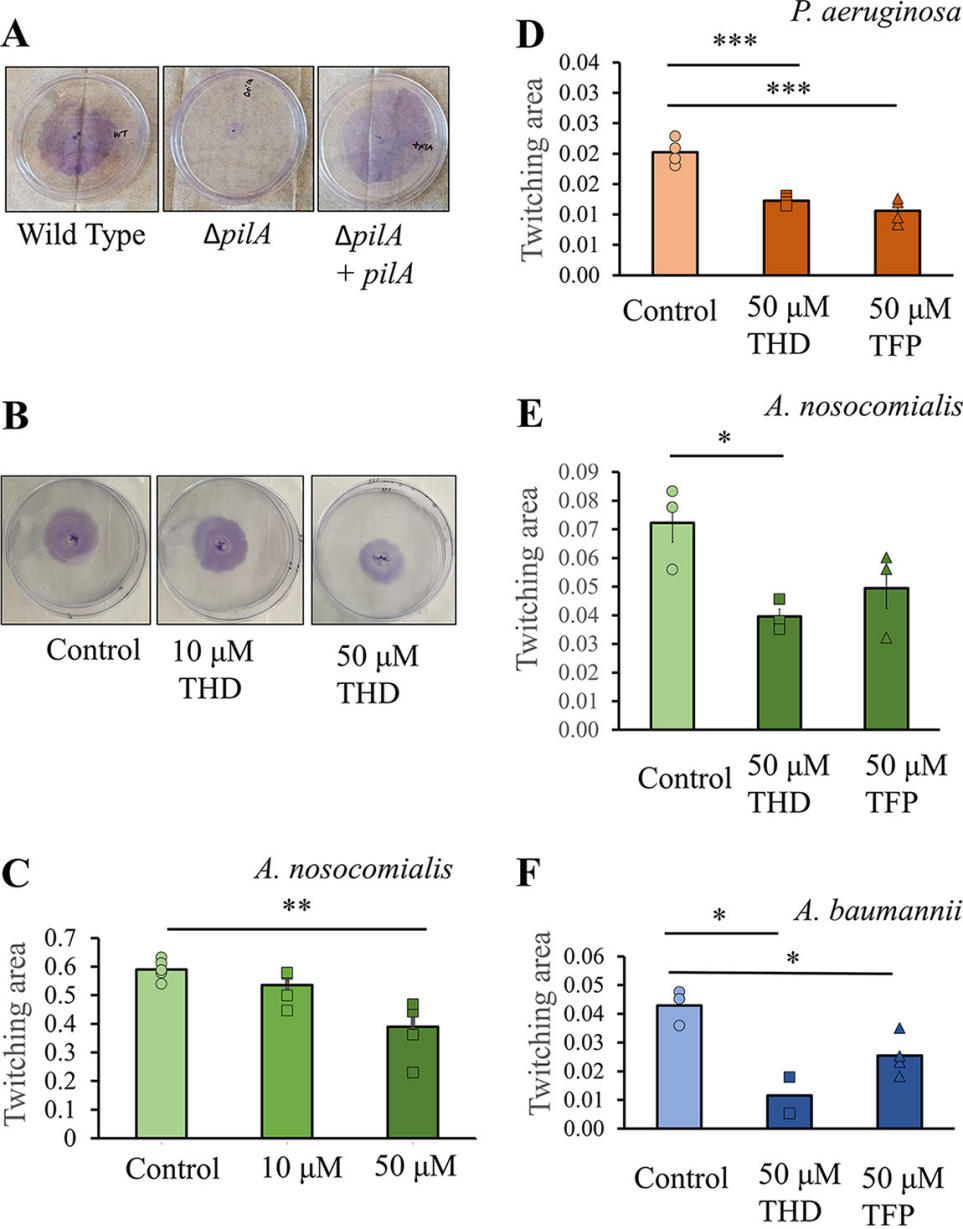
Twitching motility. (A) Representative twitching from plates inoculated with A. nosocomialis M2 wild type, Δ*pilA*, and Δ*pilA* complemented chromosomally with *pilA*. (B) Representative twitching from A. nosocomialis M2; control plates and those with 10 and 50 μM thioridazine. (C) Twitching area as a function of THD concentration. (D to F) Comparisons of twitching inhibition from THD (squares) and TFP (triangles) are shown for (D) P. aeruginosa PAO1, (E) A. nosocomialis M2, and (F) A. baumannii ATCC 17978. *, *P* < 0.05; **, *P* < 0.01; ***, *P* < 0.001.

To measure the effects of phenothiazines on T4P activity, we used a twitching motility assay. Twitching motility is surface-based, first observed in A. calcoaceticus (19–21), and can occur on semisolid surfaces or at the interface of solid and semisolid surfaces. Unlike in some other forms of surface motility, T4P is essential for twitching motility, which requires both T4P biogenesis and retraction, allowing it to be used as a proxy for T4P biogenesis and function. Fig. 2 shows twitching assays for A. nosocomialis wild type, Δ*pilA* (knockout of the major pilus subunit, unable to produce type IV pili), and a chromosomal complement (Fig. 2A) A. nosocomialis twitching at 0, 10, and 50 μM THD (Fig. 2B and C), as well as comparisons of A. nosocomialis, A. baumannii, and P. aeruginosa twitching with the addition of either TFP or THD at 50 μM (Fig. 2D to F). As described in Materials and Methods, twitching was measured at the interface between low-percentage MacConkey agar (1%) and the solid plastic surface of the petri dish. While results were similar for both compounds against *P. aeruginosa* and A. baumannii, only THD showed a statistically significant effect against A. nosocomialis; this effect was dose-dependent, with a significant (*P* = 0.0025) reduction at 50 μM, but not at 10 μM.

The precise mechanism of this inhibition remains unclear. Denis et al. hypothesized that phenothiazines inhibit microcolony formation in *Neisseria* by disrupting sodium transport across the inner membrane and showed that increasing NaCl concentrations can counteract the dispersal effect (25). However, microcolony formation in *Neisseria* appears to be largely independent of NaCl concentration. Based on prior results from other studies, we hypothesized that in Acinetobacter species, T4P function would decrease with increasing salinity (36). Figure S2 shows the effects of increasing salinity on T4P function in A. baumannii and P. aeruginosa; this assay is identical to that shown in Fig. 2, with NaCl added to the MacConkey agar medium at final concentrations of 100 and 200 mM. While no significant differences were observed between the three NaCl concentrations for P. aeruginosa, A. nosocomialis showed a clear optimum at 100 mM NaCl, with a significant reduction in twitching at 200 mM. The dependence of Acinetobacter T4P activity upon salinity implies that sodium transport is insufficient to promote T4P biogenesis and that other inhibitory mechanisms are upregulated at higher NaCl concentrations.

### Static biofilm formation assays show reduced biofilm formation for *Acinetobacter* and *Pseudomonas* treated with phenothiazines.

Previously, our group showed that Acinetobacter mutant strains lacking type IV pili were deficient in static assays of biofilm formation (11). Similar results have been shown in P. aeruginosa for T4P-deficient mutants (37, 38), and hyperpiliated mutants of P. aeruginosa show increased biofilm formation (39). Denis et al. also showed dispersal of P. aeruginosa biofilms at 100 μM TFP (25). Based on this established relationship and the results described above, we hypothesized that phenothiazine treatment would similarly inhibit biofilm formation by Acinetobacter.

**FIG 3:**
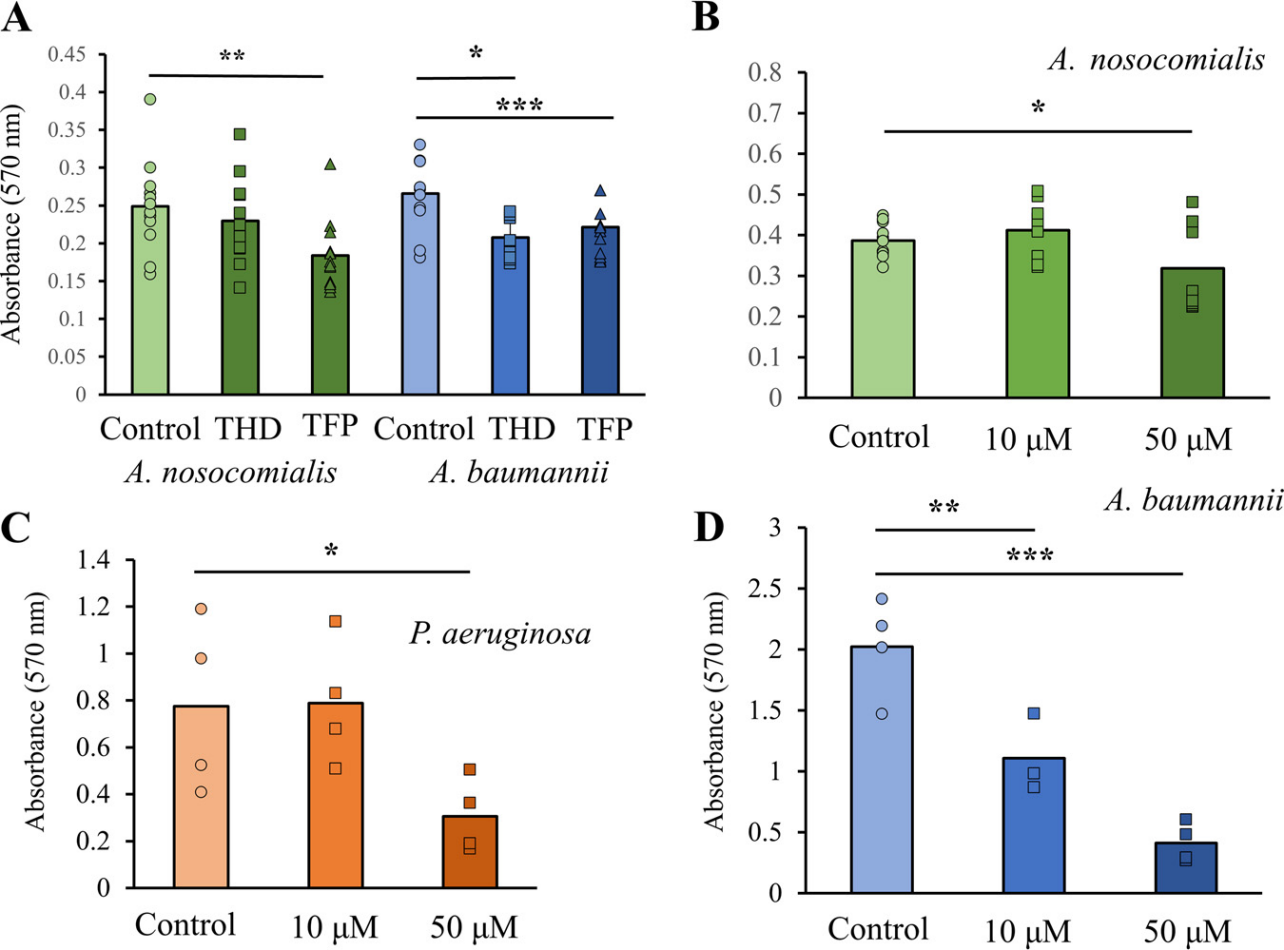
Static biofilm formation. (A) Static biofilm assay showing the effects of 50 μM TFP (triangles) and THD (squares) for A. nosocomialis M2 and A. baumannii 17978. (B to D) Static biofilm formation as a function of THD concentration is shown for (B) A. nosocomialis M2, (C) P. aeruginosa PAO1, and (D) A. baumannii 17978. *, *P* < 0.05; **, *P* < 0.01; ***, *P* < 0.001.

Figure 3A shows the results of static biofilm assays for A. nosocomialis M2 and A. baumannii ATCC 17978 with the addition of 50 μM THD or TFP. As described in Materials and Methods, these biofilms were grown in plates with minimal shaking at 37°C and the planktonic cells were removed, washed, and stained with crystal violet (40). At this concentration, both compounds significantly reduced biofilm formation in A. baumannii; however, in A. nosocomialis, only the TFP result was statistically significant, reversing the trend seen for A. nosocomialis twitching motility. Titrations of THD showed a dose-dependent effect in A. baumannii (Fig. 3D), but only the 50-μM concentration was effective against P. aeruginosa PAO1 and A. nosocomialis *M2* (Fig. 3C and D). Taken together, these results support an inhibitory effect for phenothiazines, particularly thioridazine, on Acinetobacter biofilm formation, with more consistent inhibition seen for A. baumannii ATCC 17978 (a historical lab strain) than for A. nosocomialis M2 (a more recent isolate). However, the high level of variability makes it difficult to gauge the species-specificity of each compound.

Because polysaccharides make up a portion of the extracellular matrix of bacterial biofilms (9), the formation of bacterial biofilm can result in the selective uptake of dyes which adhere to polysaccharides. Assays of Congo red uptake have been used as a measure of biofilm formation in P. aeruginosa (41). We measured the uptake of Congo red by several Acinetobacter species, with and without the addition of THD, and did not find significant effects for any species or strain of Acinetobacter despite considerable natural variation between species (Fig. S3). The insensitivity of this assay to phenothiazine compounds could indicate that biofilm formation and polysaccharide production are less correlated in Acinetobacter species than in P. aeruginosa or that biofilm formation on agar medium is less dependent upon T4P function.

### Continuous flow model of biofilm formation shows that T4P-mutants of *A. nosocomialis* are deficient in biofilm formation.

To evaluate the effect of phenothiazines on a more robust assay of Acinetobacter biofilm formation, we used a continuous flow assay in which biofilms develop under continuous shear stress (42). As an initial test of the system and to ensure that Acinetobacter biofilm formation under continuous flow was equally dependent on T4P, we measured biofilm formation for A. nosocomialis M2 wild type, Δ*pilA*, and Δ*pilA* + ppilA: the wild-type strain, a knockout strain of the major pilin subunit, and its complement (11, 16, 43). These results, shown in Fig. S4, show a clear defect in the Δ*pilA* mutant which is restored in the complement.

**FIG 4:**
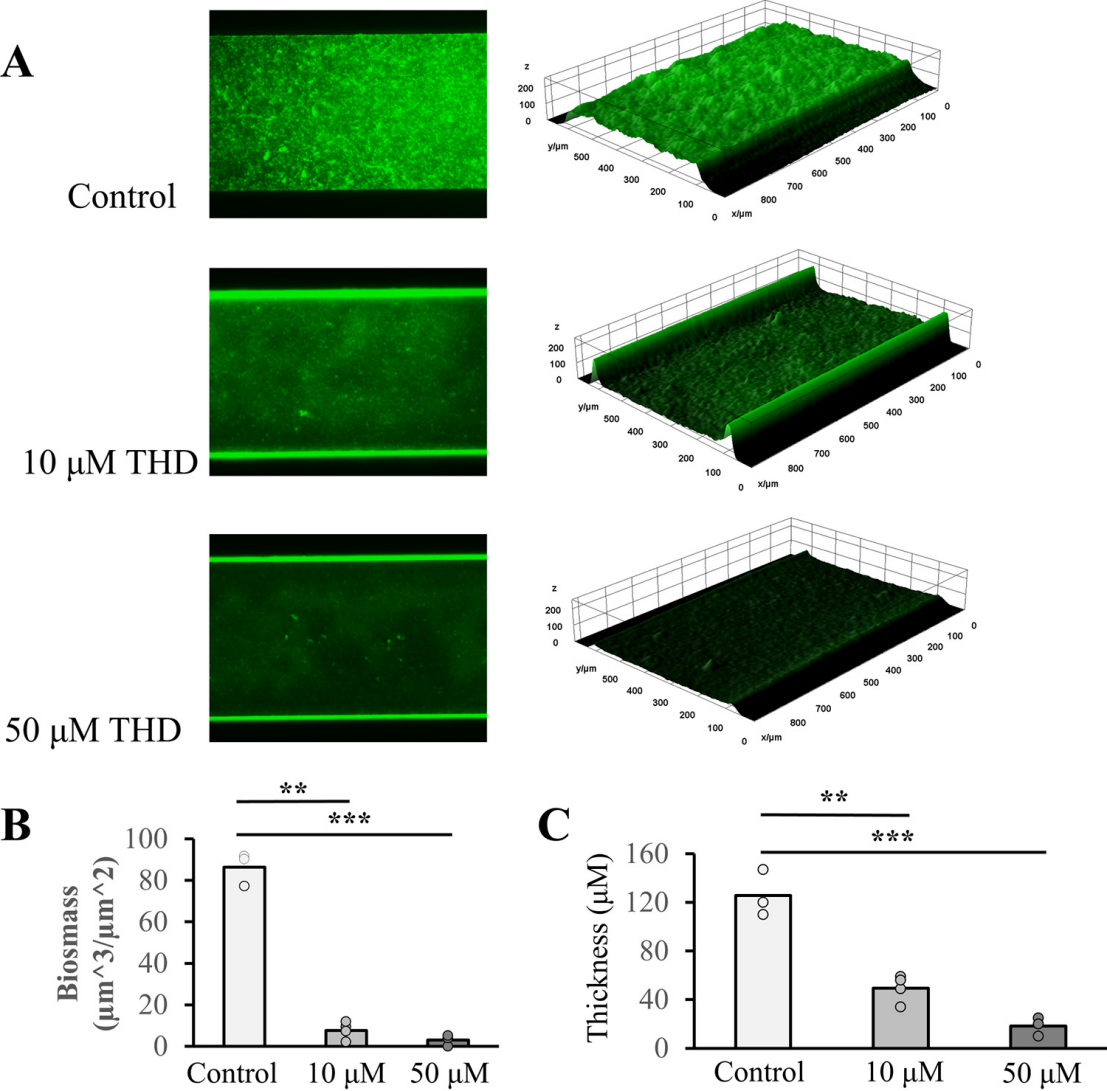
Continuous flow biofilms. (A) Representative A. nosocomialis M2 biofilms grown under continuous shear stress as a function of THD concentration. (B) Biomass as a function of THD concentration. (C) Biofilm thickness as a function of THD concentration. *, *P* < 0.05; **, *P* < 0.01; ***, *P* < 0.001; ns, not significant.

Figure 4 shows the results of continuous flow biofilm formation for A. nosocomialis with the addition of THD at 10 and 50 μM. These biofilms developed over 48 h at 37°C, as described in Materials and Methods, and were stained with a lipophilic fluorescent green dye (FM 1-43) Representative images from each flow channel are shown in Fig. 4A, along with 3D reconstructions from the z-stacks. Quantification of biomass and average thickness (calculated using Comstat 2) are shown in Fig. 4B and C. Biomass and thickness were substantially reduced at both concentrations of THD, indicating the inhibition of biofilm formation by THD is more pronounced under continuous flow than in static assays (Fig. 3B). Similar inhibition was observed for A. baumannii and P. aeruginosa when comparing bright-field microscopy images (Fig. S5).

The inhibition of biofilm formation under continuous flow was much greater than either inhibition of twitching motility or static biofilm formation. At 50 μM THD, biofilms were reduced by over 90% in terms of biomass, comparable to those of the Δ*pilA* mutant, while measured twitching motility at that concentration was still within 50% of the wild type. The strong biofilm inhibition in this model supports the potential of phenothiazine compounds for use in combinatorial therapies against Acinetobacter infections.

### Thioridazine shows activity against multiple *Acinetobacter* strains.

Although A. baumannii is commonly thought to be the cause of most Acinetobacter infections (44–46), infections with other Acinetobacter species continue to be medically relevant (1, 47–50). Considerable variation also exists within A. baumannii (51, 52), including within the T4P system (11, 53). For these reasons, we measured the ability of phenothiazine treatment to reduce biofilm formation in a range of Acinetobacter species, both within the Acb complex (A. baumannii AB5075, A. baumannii ATCC 17978, and A. nosocomialis M2) and outside it (*A. radioresistens* FO-1 and A. baylyi ADP1). All Acb members are known pathogens, but they vary in their period of isolation; A. baumannii ATCC 17978 is a well-characterized lab strain (54), while AB5075 and A. nosocomialis M2 are more recent clinical isolates (16, 55). FO-1 and ADP1 are the type strains for their respective species and are known for their extremotolerance (56, 57) and natural transformability, respectively (58, 59). Importantly, all of these species and strains produce type IV pili, suggesting that an anti-T4P inhibitory compound should be effective against all of them.

**FIG 5:**
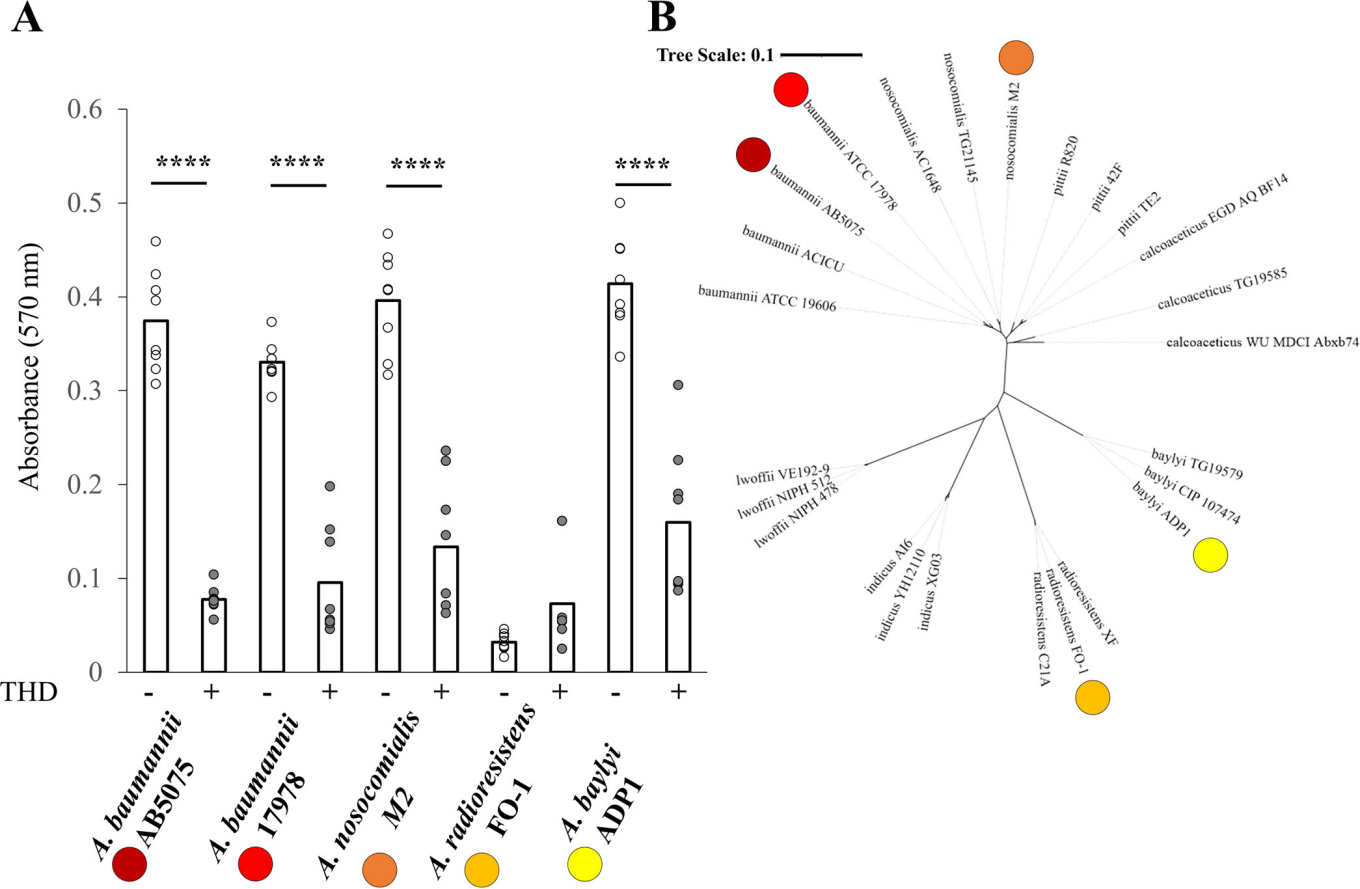
Broad THD activity against Acinetobacter biofilms. (A) Static biofilm assays for five Acinetobacter strains with and without 50 μM thioridazine. (B) Dendrogram of selected Acinetobacter strains based on the 16S rRNA methyltransferase RsmB. Strains used in panel A assay are marked with corresponding colored dots. *, *P* < 0.05; **, *P* < 0.01; ***, *P* < 0.001; ****, *P* < 0.0001.

Figure 5 shows the results of a static biofilm assay for each of the five strains with and without the addition of 50 μM THD (Fig. 5A), as well as a 16S dendrogram showing the genetic relationships between these species and strains. Apart from *A. radioresistens* FO-1, which grew robustly under both conditions but formed very little biofilm, all strains showed significant reductions in biofilm as measured by crystal violet staining.

## DISCUSSION

Although numerous physiological processes contribute to antimicrobial resistance, including drug efflux pumps and mutations to target gene products, bacterial biofilm formation remains a key factor because biofilms can persist through treatment even when isogenic planktonic cells are vulnerable (60–65). This resistance has been hypothesized to result from both the physical encapsulation and isolation from the environment (66) and the phenotypic differentiation within the biofilm making some cells metabolically inert or otherwise less vulnerable to antimicrobials (67). Biofilm inhibition or dispersal agents are an obvious solution but have been limited in their medical use by a variety of factors, including the wide variation within bacterial species which limits the utility of many specific agents (68).

Phenothiazines represent an intriguing opportunity as anti-biofilm agents because of their established activity against multiple bacteria taxa (25, 31, 34). The broad antimicrobial activity of phenothiazines, particularly thioridazine, is likely an integrated effect from numerous targets in bacterial cell membranes. However, data from our group and others suggest that inhibition of type IV pili and related filaments may be a common factor. Type IV pili are produced by a wide range of bacteria, including N. meningitidis (23, 69), P. aeruginosa (7, 19, 38, 70), and Acinetobacter spp. (11, 43), while M. tuberculosis and S. aureus produce structurally related tad/flp pili (71, 72) and competence (com) pili (73), respectively. Both T4P and com pili are known to bind DNA, while both T4P and extracellular DNA (eDNA) are known to be essential for bacterial biofilms in numerous species (8, 11, 37, 74–78). The results from studies of mutants lacking DNA-binding subunits suggest that pilus/DNA interactions stabilize biofilms (79). Here, we report the activity of two phenothiazine compounds against Acinetobacter biofilm and twitching motility at concentrations that do not show substantial inhibition of growth. The simplest explanation for these results *in toto* is that THD and TFP inhibit Acinetobacter biofilm by targeting T4P.

To the extent that activity against helical filaments like T4P can explain biofilm inhibition, the mechanism by which phenothiazines inhibit T4P function is of general interest. Previously, Denis et al. found that TFP or THD reduced piliation in N. meningitidis, but not expression of *pilE* (the homologue of Acinetobacter *pilA*), suggesting that both compounds inhibit pilus biogenesis rather than the expression of pilus subunits (25). Our results are consistent with this hypothesis, as we observed defects in both retraction-dependent twitching motility (Fig. 2) and retraction-independent biofilm formation (Fig. 3 and 4).

Experimentally, multiple models of bacterial biofilm formation are commonly used, including horizontal (80) and vertical (11) models of static biofilm formation. Continuous flow bioreactors, including the BioFlux system used here, have advantages both in the constant removal of nonadherent cells and the application of shear stress to the extracellular matrix (42, 81). Despite extensive literature showing defects in biofilm formation for bacterial mutants of type IV pilus genes (78, 82, 83), the is the first study, to our knowledge, which has measured this defect in a continuous flow model (Fig. S4 in the supplemental material). Compared directly, the defect in biofilm formation induced by THD is more pronounced under continuous flow than in static assays, particularly at a lower concentration (10 μM) (compare Fig. 3B with Fig. 4B and C). This difference is consistent with previous data showing greater differences in biofilm formation in continuous flow assays (5).

If the antimicrobial activity of phenothiazines stems, either wholly or partially, from a decrease in biofilm mediated by inhibition of pilus biogenesis, this could explain why the most dramatic results have been obtained through combined treatments in which phenothiazines potentiate the use of other antimicrobials (31, 33). Biofilm dispersal agents have been shown to potentiate other antimicrobials against numerous bacterial species (84).

Type IVa pilus systems like those found in Acinetobacter, Pseudomonas, and other *Moraxellaceae* species are involved in multiple potentially pathogenic processes, including host-cell adhesion, surface motility, horizonal gene transfer, and biofilm formation, leading to their moniker of “prokaryotic Swiss army knives” (82). This widespread activity makes them natural therapeutic targets and the use of anti-T4P compounds has precedent in Acinetobacter; Nait Chabane et al. found that Virstatin, a T4P-inhibitor, could reduce both motility and biofilm formation (54). The potential for phenothiazine use against Acinetobacter infections may depend on the strain and location of the infection. Results from several epidemiological studies suggest that A. baumannii lineages are preferentially associated with certain types of infections. Matsui et al. found that European clone II isolates made up 43% of lung infections but 0% of blood infections and only 2% of other infections (85), and Eijkelkamp et al. found that European clone II stains formed 70% more biofilm than other lineages (51). Vijayakumar et al. compared the biofilm formation capacity of lung and blood isolates and found that lung isolates formed more biofilm (approximately twice as much) on average (86). Differential roles for biofilm based on the infection site may explain why Wang et al. found no relationship between biofilm formation from clinical isolates and clinical outcomes in patients with bloodborne infections (bacteremia) (87). Taken together, these results suggest that biofilm dispersal agents (including phenothiazines) in Acinetobacter treatment may be best used for persistent lung infections, while bloodborne infections require other tools.

The broad activity observed here against biofilm formation in several Acinetobacter species (Fig. 5) is encouraging, particularly taken together with prior studies of distantly related bacterial taxa. However, we expect that phenothiazines likely have pleiotropic effects both against Acinetobacter and other genera. The cytotoxic activity we observed against *M. bovoculi* cannot be explained by activity against any function of T4P, which is not essential for growth in broth (and, in fact, is generally poorly expressed in liquid culture). In humans, phenothiazine compounds are known to have activity against a wide range of membrane receptors (88) and, based on the combination of effects observed here (both anti-piliation and cytotoxicity), we expect that multiple bacterial membrane receptors are potential targets. Effective evaluation of specific activities, such as the inhibition of type IV pilus biogenesis, will require careful controls as well as specific assays (for example, twitching motility is specific for type IV pili, while many surface structures can contribute to general surface motility).

As the level of multidrug resistance in Acinetobacter infections increases, resistance is being encountered even to “last-resort” antibiotics such as colistin (89, 90). The development of combinatorial treatments which combine existing compounds approved for human use may be the most promising path forward for combating novel antimicrobial resistance as it develops. Several phenothiazine compounds have extensive histories of use in humans, including chlorpromazine, perphenazine, prochlorperazine, and promethazine, in addition to the two used in this study, thioridazine and trifluoperazine (26, 29). However, their use has been limited in some cases due to their toxicity, including ocular toxicity (91). Because courses of antibiotic treatment are generally short, the side effects of phenothiazine compounds may be less of a concern for their use as antimicrobials than for their use as anti-psychotics. The broad activity against biofilm formation by Acinetobacter species shown here for thioridazine suggests that phenothiazine compounds will be useful in combating Acinetobacter antimicrobial resistance where it is mediated by biofilm. These results, in combination with those from studies of other bacteria, suggest that similar results may be obtained for a range of bacterial species which utilize helical pili to form biofilm.

## MATERIALS AND METHODS

### Bacterial strains.

A. nosocomialis wild type, Δ*pilA* mutant, and the complement have been described previously (16, 43). A. baumannii AB5075-UW was purchased from the University of Washington’s AB5075 mutant library (92). P. aeruginosa PAO1, A. baylyi ADP1, A. baumannii ATCC 17978, and *A. radioresistens* FO-1 were purchased from the American Type Culture collection (ATCC). M. bovoculi 58086 was provided by Dustin Loy’s group at the University of Nebraska-Lincoln.

### Phenothiazine compounds.

Thioridazine (also known as Novoridazine) and trifluoperazine (also known as Stelazine), purchased from Tokyo Chemical Industry Co., Ltd. (TCI) America, were dissolved in double-distilled water (ddH_2_O) at stock concentrations of 10 mM and stored frozen in 1-mL aliquots at −20°C. Aliquots were thawed and added to bacterial growth medium (LB or MacConkey) at working concentrations of 10 to 50 μM immediately before use. For solid media, THD or TFP was added to the agar after cooling to 50°C as the plates were poured and thoroughly mixed before the agar solidified.

### Planktonic growth.

Growth in liquid medium was measured by optical density at 600 nm using a Synergy H1 (BioTek Instruments) plate reader at 37°C in 96-well plates with MacConkey broth (Difco) (A. nosocomialis and P. aeruginosa) or brain heart infusion (BHI) broth (Remel) (*M. bovoculi*). Overnight cultures of each bacterial species were added at 1:10 and growth measured for at least 6 h with periodic shaking for equilibration (93).

### Twitching motility.

For all bacterial strains, bacteria were grown on solid medium (LB agar at 1.5%) overnight at 37°C. For measuring twitching motility, assay plates were made using MacConkey medium with 1.0% agar (previously found by our group to increase twitching in Acinetobacter species [11]). Multiple colonies were removed from a single plate grown overnight and inoculated into the assay plates by stabbing through the agar to the interface between the medium and the petri dish. Assay plates were incubated at 37°C for at least 48 h in sealed bags with moist paper towels (to control humidity for the duration of the assay). Twitching was measured by removing the agar medium from the petri dish and staining the bacteria adherent to the petri dish with 0.1% crystal violet in phosphate-buffered saline (PBS; 20 mM NaPO_4_, 150 mM NaCl [pH 7.4]). After staining, petri dishes were washed with PBS to remove nonspecific crystal violet and twitching area was measured in two directions 90 degrees apart as described previously (11, 94). Total twitching area was measured as a fraction of the plate’s total area calculated from the radius of the plate in the image.

### Static biofilm formation.

Static biofilm formation was measured after growth in LB medium without added NaCl (5 g yeast extract and 10 g peptone per L). Next, 96-well plates were inoculated 1:20 with bacteria grown overnight at 37°C. Plates were then incubated at 37°C for 72 h, with liquid medium exchanged after 24 and 48 h. Wells were then washed 3 times with PBS, stained with 0.01% crystal violet for 10 min, and washed three more times with PBS. Crystal violet was dissolved in 30% acetic acid and measured (after dilution) by absorbance at 550 nm using a Synergy H1 plate reader (BioTek Instruments) (5). Similar results were obtained using MacConkey medium (Fig. S6).

### Biofilm formation under continuous flow.

Bacterial biofilms were grown in a 1:5 dilution of MacConkey medium, under continuous shear stress, in a BioFlux 200 (Fluxion Biosystems) over 48 h at a flow rate of 1 μL/min. Progression was monitored using bright-field microscopy with an EVOS m5000 (Thermo Fisher). For imaging, biofilms were fluorescently stained for 5 min using FM 1-43 (Thermo Fisher), a green fluorescent lipophilic dye, at 4 mM in PBS, and washed using 50 μL PBS (20 mM NaPO4, 150 mM NaCl [pH 7.4]) at 5 μL/min, similar to previous studies (79, 95). The resulting fluorescent bacterial biofilms were imaged using the EVOS m5000; z-stacks were taken, and the resulting 3D composite images were analyzed for biomass and thickness using Comstat 2 (96) and ImageJ (97).

### Congo red/brilliant blue uptake.

This assay was performed as described previously (41). Briefly, solid medium for these plates consisted of 15 g/L peptone, 10 g/L lactose, 1.5 g/L bile salts, 13.5 g/L agar, 40 mg/L Congo red, and 20 mg/L Coomassie brilliant blue. Bacterial suspensions were spotted in replicates on multiple plates grown for 24 h at 37°C. The images were analyzed using ImageJ (97), with the intensity of the blue channel used to determine the density of dye for each colony.

### Statistical analysis.

All experiments included at least three technical replicates. Statistical analyses were performed using R for Windows 3.6.1; comparisons between groups used Student’s one-tailed *t* test.
