# Supplementary material for: Type IV Pilus-Mediated Inhibition of Acinetobacter baumannii Biofilm Formation by Phenothiazine Compounds
Source: Microbiol Spectr. 2023 Jun 21;11(4):e01023-23. doi: 10.1128/spectrum.01023-23 (PMC10433872; doi:10.1128/spectrum.01023-23)
Supplement: Supplemental file 1 — Supplemental material. Download spectrum.01023-23-s0001.pdf, PDF file, 0.9 MB [file spectrum.01023-23-s0001.pdf]

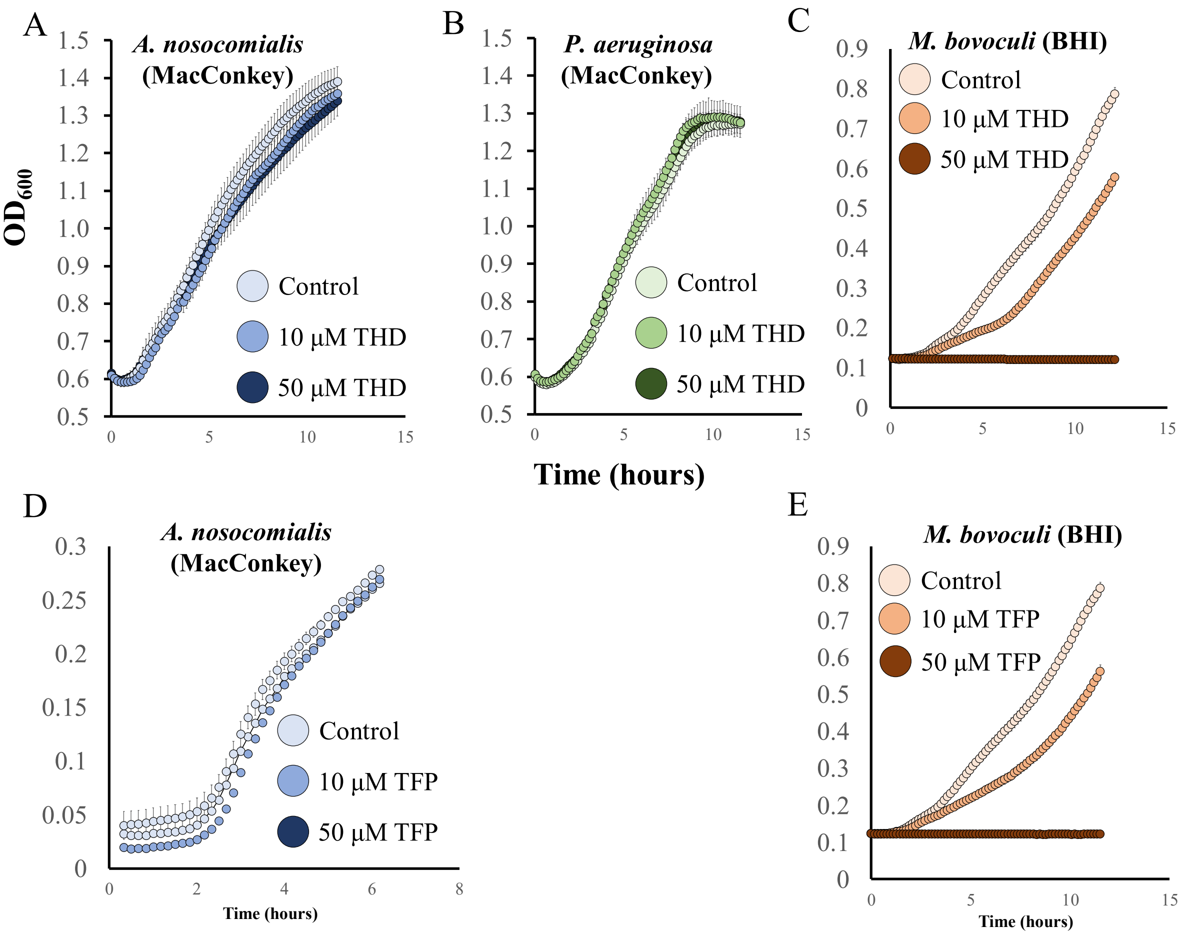

Supporting Figure 1: Planktonic growth at micromolar concentrations of Thioridazine and Trifluoperazine. *A. nosocomialis* M2 and *P. aeruginosa* PAO1 were grown MacConkey medium (DifCo) while *M. bovoculi* 58086 was grown in Brain-Heart Infusion (BHI) medium (RPI). THD (panels A-C) or TFP (panels D and E) were added at either 10 or 50 µM. In all cases, growth curves were measured starting from 1:10 dilutions of overnight cultures.

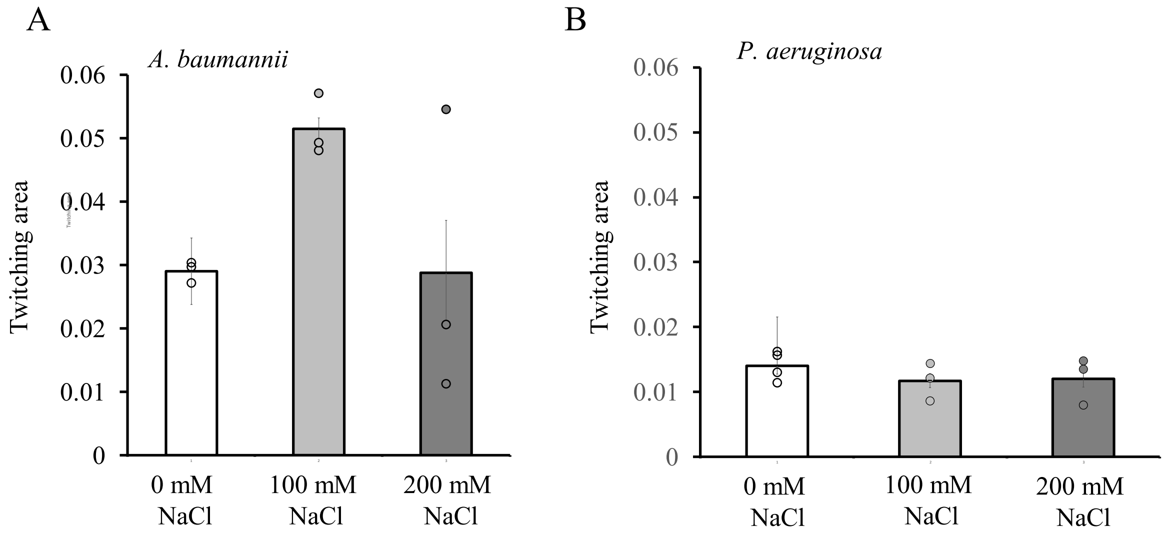

Supporting Figure 2: Twitching motility as a function of NaCl concentration. Twitching motility was measured as described in Methods using 1% MacConkey agar over 24 hours.

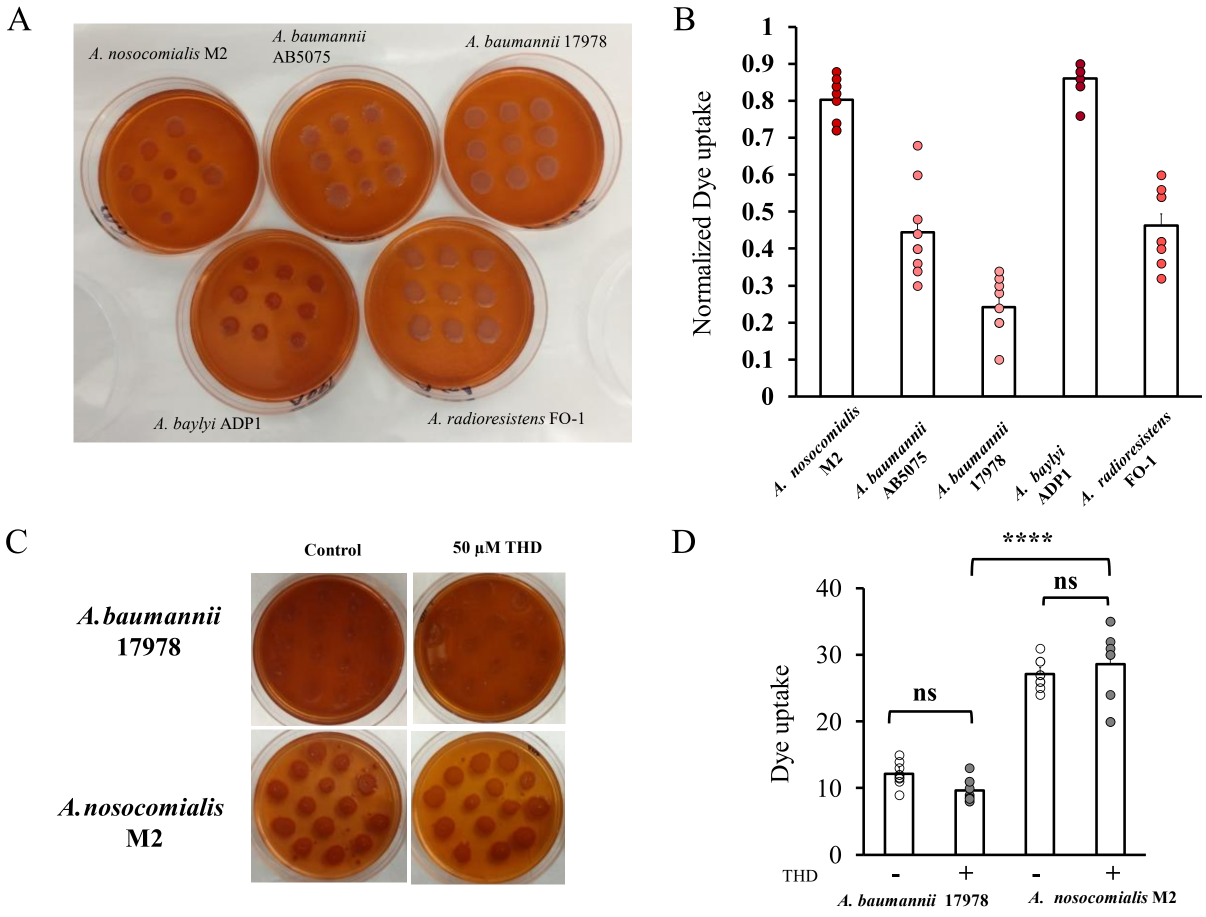

Supporting Figure 3: Congo Red Dye uptake. A) Dye uptake for five strains of *Acinetobacter*. B) Dye uptake from A) as measured by color analysis. C) Dye uptake by *A. baumannii* and *A. nosocomialis* with and without the addition of Thioridazine. D) color analysis of C).

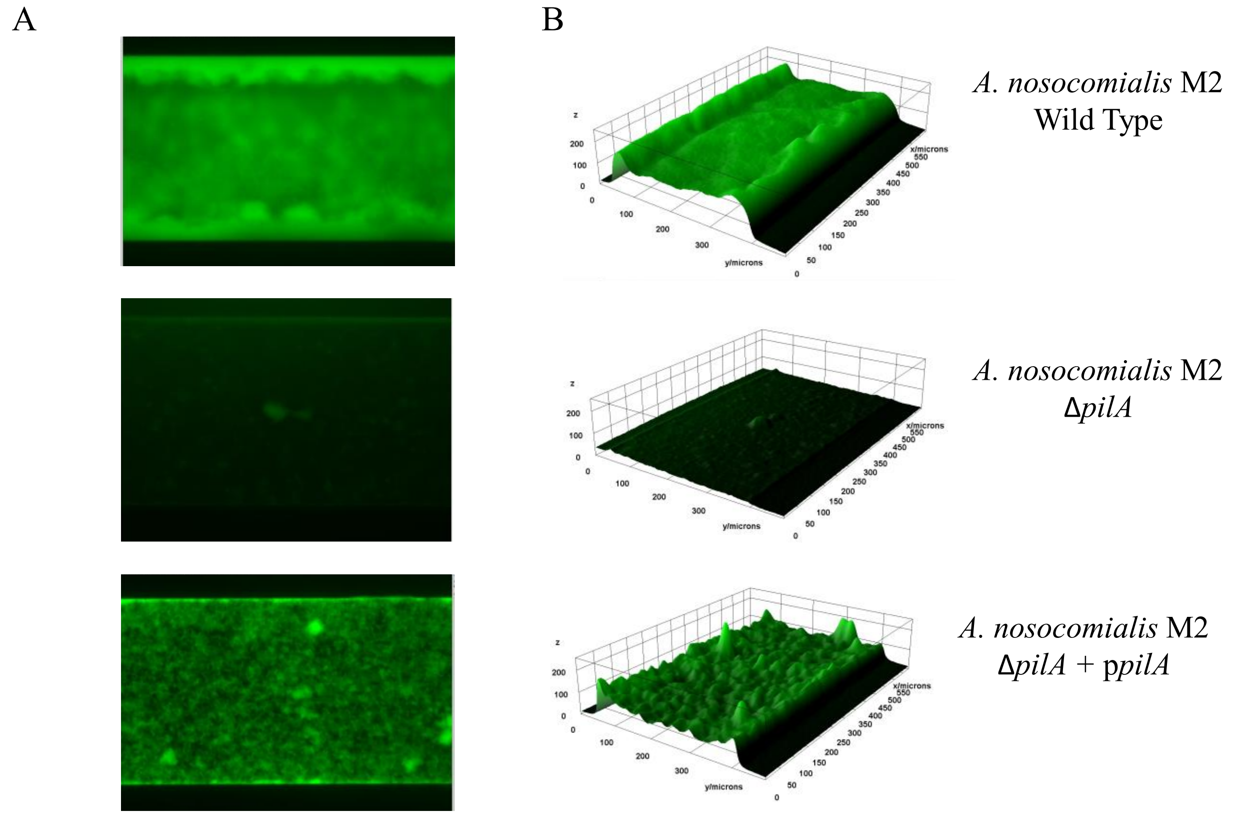

Supporting Figure 4: Biofilm formation of T4P mutants under continuous flow. Representative images are shown for flow cells (top down) (A) and 3D reconstructions (B) for A. nosocomialis M2 wild type, Δ*pilA* (the major T4P subunit) and its complement. Continuous flow biofilms were grown and stained as described under methods.

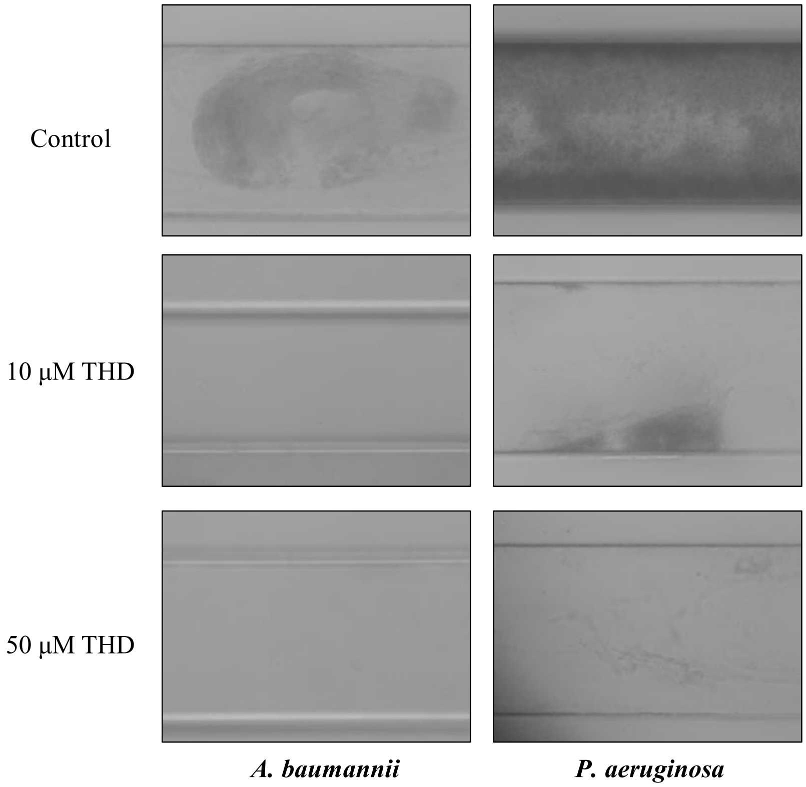

Supplementary Figure 5: Brightfield images of biofilms under continuous flow. Biofilms were grown as described under methods. Images were taken with an EVOS m5000 with a phase contrast objective.

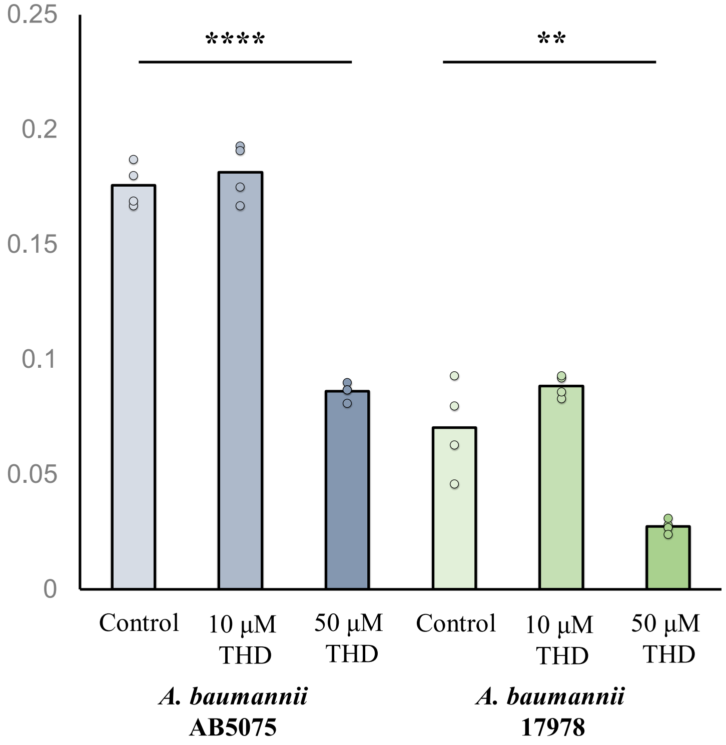

Supplementary Figure 6: Static Biofilm formation in MacConkey Medium. Static biofilms of *A. baumannii* in 96-well plates were grown as described in Methods, save that rather than Luria broth, MacConkey medium was used.
